# Supplementary figures and images for: Synergistic effects of anti-PDL-1 with ablative radiation comparing to other regimens with same biological effect dose based on different immunogenic response
Source: PLoS One. 2020 Apr 14;15(4):e0231507. doi: 10.1371/journal.pone.0231507 (PMC7156084; doi:10.1371/journal.pone.0231507)

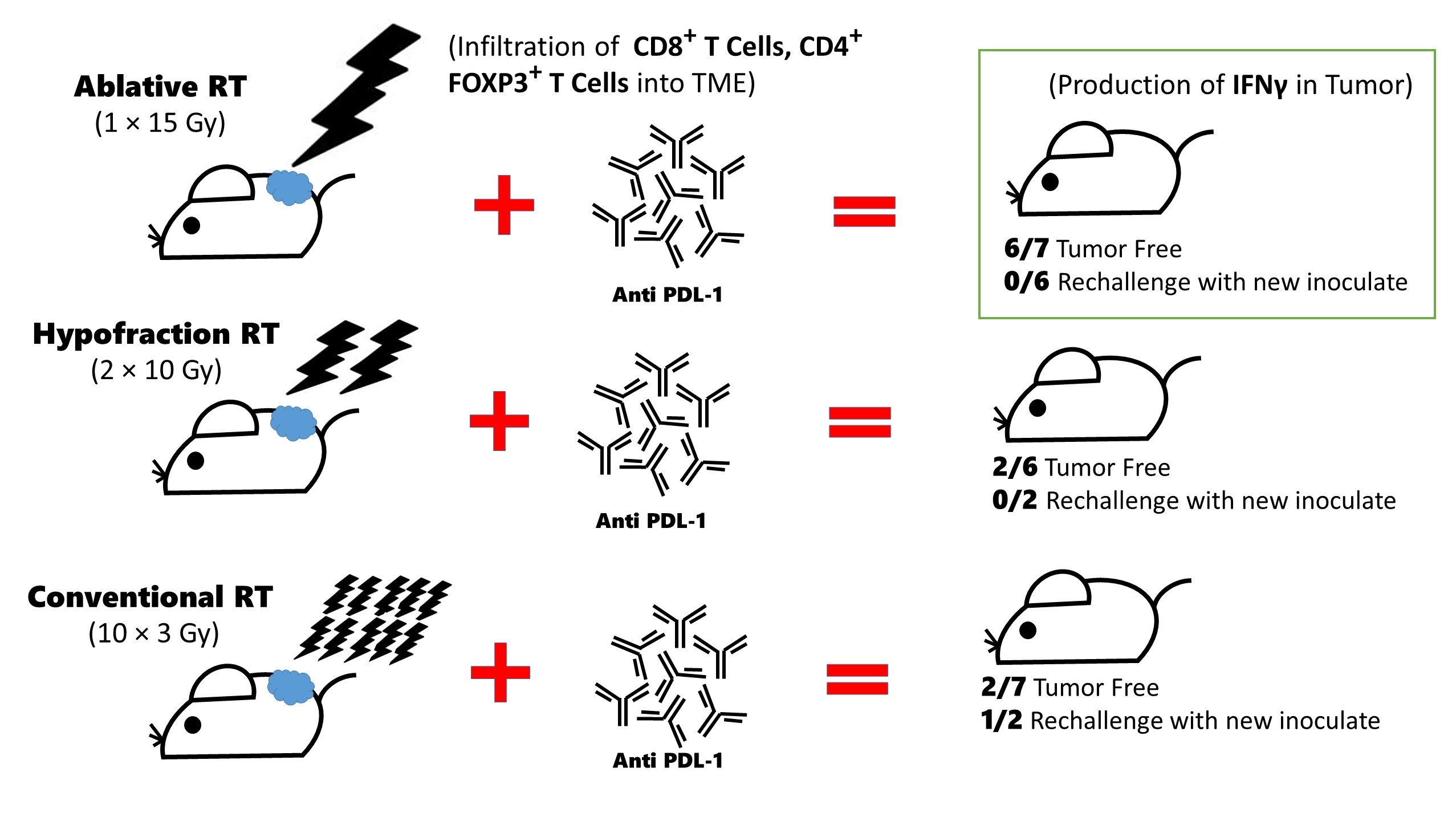

Supplement: S1 Fig — (TIF) [file pone.0231507.s002.tif]
